# Emerging applications of exosomes in cancer therapeutics and diagnostics

**DOI:** 10.1002/btm2.10059

**Published:** 2017-04-03

**Authors:** Sahil Inamdar, Rajeshwar Nitiyanandan, Kaushal Rege

**Affiliations:** ^1^ Chemical Engineering Arizona State University Tempe AZ 85287; ^2^ Biological Design Program, Arizona State University Tempe AZ 85287

**Keywords:** drug delivery, immunotherapies, nanobiology, patient‐targeted therapies

## Abstract

Exosomes are nanoscale extracellular vesicles that are shed from different cells in the body. Exosomes encapsulate several biomolecules including lipids, proteins, and nucleic acids, and can therefore play a key role in cellular communication. These vesicles can be isolated from different body fluids and their small sizes make them attractive in various biomedical applications. Here, we review state‐of‐the art approaches in exosome isolation and purification, and describe their potential use in cancer vaccines, drug delivery, and diagnostics.

## 1 Introduction

**Figure 1:**
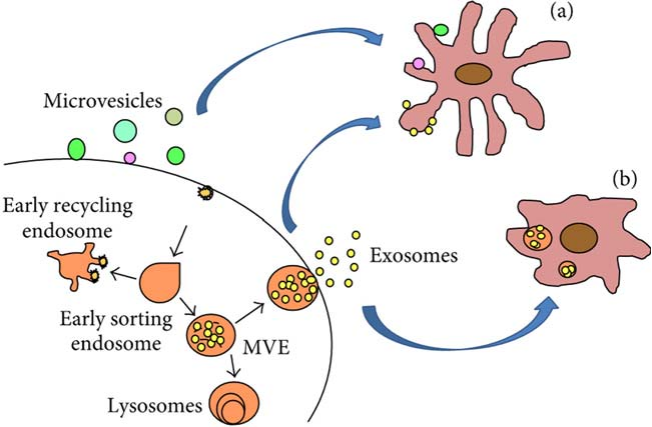
Schematic showing the release of exosomes and other vesicles from cells. Adapted from Ref. 8

Communication between cells is a critical process that occurs in all organisms. This sharing of information is facilitated either horizontal gene transfer by viruses^1^ and bacteriophages^2^ or by the secretion of soluble factors.^3^ Cells secrete extracellular vesicles (EVs), namely, apoptotic bodies, microvesicles, and exosomes,^4^ which can have significant impact on the local microenvironment as well as on distant tissues in the body.^5^ Apoptotic bodies are the largest among EVs, with sizes ranging from 50 to 5,000 nm, and contain DNA, RNA, and histone proteins. Apoptotic bodies are eventually removed by macrophages.^6^, ^7^ Microvesicles (50–1,000 nm) are generally smaller than apoptotic bodies and are also known as shedding vesicles or exovesicles. Exosomes (30–120 nm; Figure 1),^8^ are the smallest among secreted vesicles, and consist of a lipid bilayer membrane that surrounds cytosol and other contents. Exosomes derived from human embryonic kidney cells have been observed to shrink in size when stored under 4°C temperatures.^9^ Exosomes typically do not contain cellular organelles but can carry different molecular constituents, including proteins and nucleic acids, from the cell of origin.^10^ Proteins including those from the tetraspanin family (CD9, CD63, CD81, and CD82), ESCRT complex (TSG101, Alix)^11^ and heat shock proteins (Hsp 60, Hsp70, Hsp90) are known to be found in exosomes; the composition of these proteins differs based on the cell or tissue of origin.^12^, ^13^

Vesicles secreted by cells can meet one of the following fates: (a) internalization by other cells in the immediate proximity, (b) internalization by cells at a significant distance away from the cell of origin, and (c) removal by distant tissues following entry into systemic circulation.^14^ Exosomes carry proteins and nucleic acids from their cell of origin,^15^ and these contents can be delivered to a different recipient cell leading to intercellular communication that can impact different physiological process.^5^, ^16^, ^17^ Western blotting^18^, ^19^, ^20^, ^21^, ^22^, ^23^, ^24^ and fluorescence‐activated cell sorting analysis^22^, ^25^, ^26^, ^27^ of beads coated with exosomes have helped determine the presence of known cellular proteins in exosome preparations from different cells. In addition, mass spectrometry can be employed to identify unknown cellular proteins present in exosomes.^27^, ^28^, ^29^, ^30^ Exosome‐facilitated messages can regulate cellular growth, division, and apoptosis;^31^, ^32^ changes in gene expression in recipient cells can be induced following delivery of multiple miRNAs by exosomes.^33^ For example, exosomes derived from three different melanoma cell lines, while being morphologically very similar, had different effects on T‐cell proliferation/growth suppression.^34^ In this review, we first discuss cell sources that have been explored for obtaining exosomes and isolation methods that have been employed for obtaining enriched populations of these vesicles. We also discuss the use of exosomes as therapeutic carriers, disease biomarkers, and vaccines.

## 2 Cell sources for exosomes

Cell lines including bEND3 and EL‐4 are predominantly used for isolation of exosomes used in drug delivery due to the ease of their availability, the ability to scale up and the ability to generate exosomes of similar quality.^35^, ^36^ Mesenchymal stem cells or hESC‐MSCs are also excellent sources for isolation of exosomes,^37^ and it has been observed that exosomes isolated from these are tolerated well by the immune system thereby making them an excellent choice for drug delivery.^15^, ^38^, ^39^ These cell lines can also be used to isolate exosomes for the purpose of developing anti‐cancer vaccines. It is possible that primary cells isolated from patients/mice for developing these exosome‐based anti‐cancer vaccines might demonstrate a batch to batch variation in strength of the immune response.^20^, ^40^ The use of exosomes for identifying biomarker levels mandates that patient‐derived cells/body fluids are used. The presence/absence of disease can be determined by comparing exosomal biomarker levels of the patient with that of healthy controls.^41^, ^42^, ^43^, ^44^, ^45^

## 3 Isolation and purification of exosomes

Several methods have been investigated for the isolation and purification of exosomes from biological fluids.^46^, ^47^ Centrifugation, filtration, immunological separation, microfluidic isolation,^48^ and size‐exclusion chromatography can be effectively applied in both laboratory research and clinical medicine. Differential ultracentrifugation remains one of the most common techniques for exosome isolation^49^, ^50^, ^51^ and consists of several centrifugation steps that remove cells, large vesicles, and debris at lower centrifugation speeds. The pellets are discarded while the supernatant is subjected to higher centrifugation speeds in order to obtain exosomes as a pellet. Differential ultracentrifugation is commonly employed for the isolation of exosomes but the efficiency of the method is lower when plasma and serum are used due to higher viscosities of these fluids.^24^, ^47^, ^52^ Density gradient centrifugation combines differential ultracentrifugation with a sucrose density gradient. This method is mainly used for separating exosomes from nonvesicular particles including proteins and protein/RNA aggregates. The method allows separation of low‐density exosomes from other contaminants but is highly sensitive to the centrifugation time.^24^, ^52^, ^53^

Specific binding of antibodies to receptors present on the surface of exosome have been explored for isolating these vesicles from mixtures^54^, ^55^; in many cases isolated exosomes are subsequently analyzed for DNA or total RNA.^56^, ^57^, ^58^, ^59^, ^60^ As an application of this approach, antibodies are displayed onto magnetic beads to facilitate the specific binding and isolation of exosomes.^61^, ^62^ The advantage of this immunoaffinity technique is the presence of various tetraspanins on exosomes isolated from different cell types which can be targeted using antibodies. The immunoaffinity technique employed was reported to be more effective at isolating exosomes compated to ultracentrifugation and density gradient methods.^53^, ^63^ This method can be extremely effective for small‐scale applications with low volume samples but the costs associated with scaling up this approach may be prohibitive for large scale isolation procedures.

Ultrafiltration has been investigated for the separation of exosomes from proteins,^39^, ^64^, ^65^ although the efficacy of this method has not been fully established for clinical samples. A porous membrane can be used for trapping exosomes resulting in their isolation from cell culture media. The filtration membrane helps concentrate the exosomal population. Recovery of exosomes from the membranes is facilitated by using ethanol or NaOH, which is typically followed by rinsing with phosphate‐buffered saline.^24^ However, application of large external force in this approach can result in deformation or damage of exosomes.

**Figure 2:**
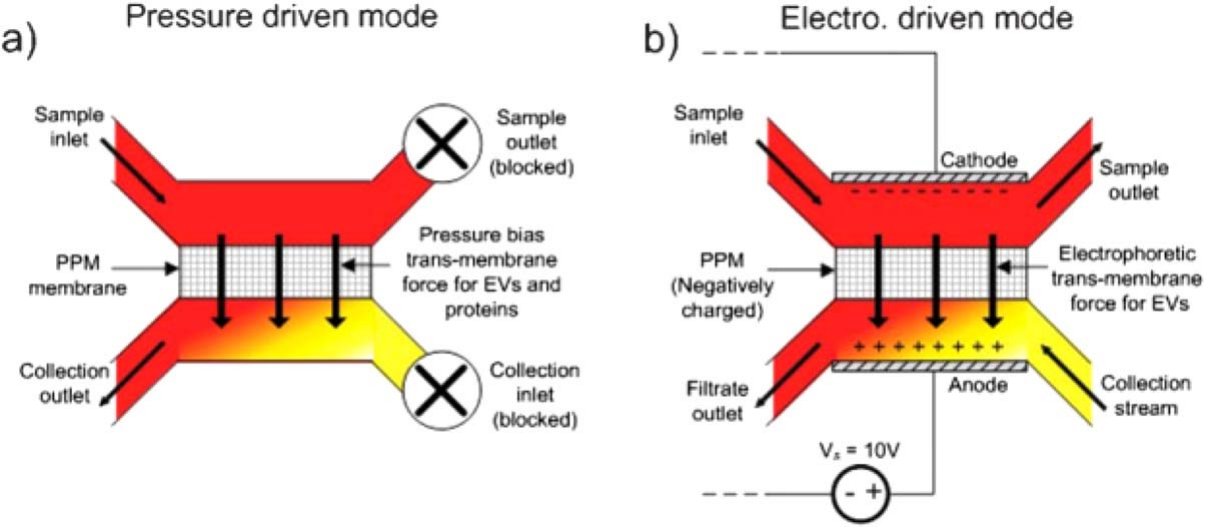
Schematic of the polymer‐based precipitation method used for the isolation of exosomes. Adapted from Ref. 54

**Figure 3:**
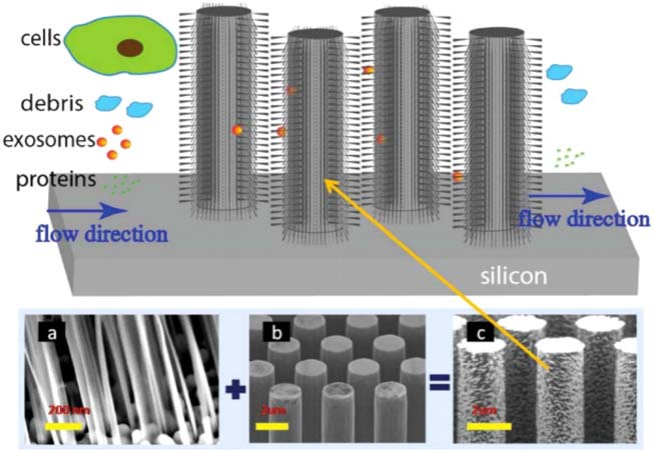
Schematic of the size‐exclusion chromatography approach employed for the isolation and purification of exosomes. Adapted from Ref. 58

Polymer‐facilitated precipitation, most commonly using polyethylene glycol, is also employed for recovering exosomes from mixtures (Figure 2).^66^, ^67^ The main advantage of this method is the use of neutral pH. Although, there are no adverse effects on the isolated exosomes, contamination of exosomes with other nonexosomal materials is a significant drawback of this method.^68^ In addition, the presence of the polymer may interfere with downstream analyses and/or usage.^69^ Polymer‐facilitated precipitation can be significantly improved by using methods for removing the polymer used in the operation. Size‐exclusion chromatography is commonly used to separate macromolecules based on size,^70^ and has also been investigated for exosome isolation (Figure 3).^71^, ^72^ Exosomes isolated using this method are subjected to minimal shear force, resulting in relatively low damage to the structure of these vesicles; however, deformation of larger vesicles has been reported.^73^

Exosomes isolated using ultracentrifugation—for example, using the ExoQuick approach—can result in increased yields compared to other methods, while also maintaining the quality of the exosomes isolated.^24^ The swift isolation and higher exosome recovery enables analysis of protein expression in the recovered exosomes. Ultracentrifugation with density gradient centrifugation can improve the efficacy of the exosome purification without damaging the morphology. Variations in sizes of isolated exosomes have also been observed between fresh samples and those stored in DMSO and freezing also resulted in degradation of exosomal RNA over time.^74^

## 4 Applications of exosomes

### 4.1 Drug and nucleic acid delivery

**Figure 4:**
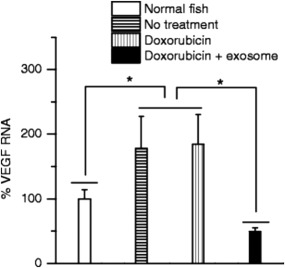
In vivo delivery of doxorubicin (Dox)‐loaded exosomes across the blood brain barrier in zebrafish model. Significant inhibition of VEGF was observed in Dox‐loaded exosomes compared to untreated controls and those treated with unencapsulated doxorubicin. Adapted from Ref. 35

The small sizes of exosomes make them attractive as vehicles in drug and nucleic acid delivery, although detailed molecular characterization of exosomes is necessary before adopting them in widespread applications. Yang et al. hypothesized that exosomes derived from brain cells displayed brain‐specific surface proteins which allowed them to pass through the blood‐brain barrier (BBB) and deliver drugs across this barrier.^35^ Exosomes were isolated from four different cell lines including brain endothelial cell line bEND.3, human glioblastoma cell line U‐87 MG, human brain neuroectodermal cell line PFSK‐1, and human brain glioblastoma A‐172 cells. Rhodamine‐123 (2 mg/ml), doxorubicin, or paclitaxel were independently loaded into different exosomes by mixing followed by incubation for 2 hr. Doxorubicin‐loaded exosomes demonstrated higher efficacies for inducing death in U87‐MG glioblastoma cells compared to paclitaxel‐loaded exosomes. Exosomes isolated from bEND.3 cells were able to deliver the rhodamine‐123 dye across the BBB following delivery via the cardinal vein of zebrafish embryos. Exosomes from bEND.3 cells were able to deliver drugs to a U‐87 MG tumor grown in the brain of zebrafish and were also observed to inhibit VEGF (vascular endothelial growth factor) levels in vivo (Figure 4).^35^ This, in turn, was able to induce a significant decrease in the tumor size, compared to treatments with the free drug. Intranasal delivery of curcumin or JSI‐124 (cucurbitacin I) inhibitor‐loaded exosomes was investigated as a potential therapeutic approach for brain inflammatory diseases.^75^ Three different mouse models exhibiting lipopolysaccharide (LPS)‐induced brain inflammation, autoimmune encephalatis, or the GL26 brain tumor model which exhibits inflammation due to infiltration of immune cells including macrophages and T‐cells, were used in the study.^76^ Exosomes were loaded by mixing curcumin with EL‐4 (mouse lymphoma cell)‐derived exosomes at a temperature of 22°C after which the loaded exosomes were separated using sucrose gradient centrifugation. Intranasally delivered exosomes were taken up by microglial cells, which are key mediators in neuro‐inflammatory diseases; delivery of curcumin‐loaded exosomes resulted in a reduction in activated microglial cells in both encephelatis and LPS‐induced brain inflammation models. Intranasal delivery of exosomes loaded with the STAT3 inhibitor JSI‐124 resulted in increased survival of mice with GL26 brain tumors. This study suggests that intranasal delivery of exosome‐encapsulated drugs could lead to a noninvasive approach for direct drug delivery to the CNS.^75^

Exosomes isolated from RAW264.7 macrophages were loaded with catalase as a potential therapeutic approach for Parkinson's disease; catalase was loaded in order to potentially degrade reactive oxygen species in order to protect from inflammation.^77^ Exosomes were loaded using four different methods—incubation with saponin, sonication, freeze‐thaw, and extrusion. Sonication‐loaded exosomes resulted in the highest uptake of catalase in PC12 neuronal cells in vitro as observed using fluorescence spectroscopy and confocal microscopy. Sonication was carried out using 20% power at 500V, 2kHz for 6 cycles pulsed for 4 s on and paused for 2 s. Extrusion was also employed for loading, and was performed by mixing catalase with exosome solution followed by 10 rounds of extrusion through a Avanti Lipids extruder.^78^ Catalase‐loaded exosomes, named exoCAT, protected neuronal cells from oxidative stress in vivo following intracranial injection into C57BL/6 mice. Biodistribution of exoCAT following intracranial injections indicated localization primarily in neuronal and microglial cells but also in astrocytes and endothelial cells.

Exosomes extracted from cow milk were employed for the delivery of therapeutic molecules against lung and breast cancer.^79^ Exosomes loaded with withaferin‐A were injected intraperitoneally into female athymic nude mice subcutaneously injected with A549 cells. A tumor inhibitory effect was observed with withaferin‐A loaded exosomes at doses lower than those observed with the unencapsulated drug.

Exosomes isolated from dendritic cells from the bone marrow of C57BL/6 mice were employed for delivering small interfering RNA (siRNA) to the brain. A Rabies viral glycoprotein (RVG) peptide (single letter amino acid sequence: YTIWMPENPRPGTPCDIFTNSRGKRASNG) was displayed onto the exosomal surface for targeting the acetylcholine receptor in the brain. Electroporation at 400 V and 125 μF was used to load these exosomes with siRNA against GAPDH, and the loaded vesicles were employed to investigate the knock down of GAPDH gene in C2C12 (murine muscle) and Neuro2A (neuronal cells). Gene knockdown efficacy using exosomes loaded with GAPDH siRNA was similar to that observed using lipofectamine. Exosomes were also well tolerated and did not induce strong immune responses in C57BL/6 and BALB/C mice. RVG exosomes were also able to deliver BACE1 siRNA across the BBB.^80^

Exosomes derived from human bone marrow mesenchymal stem cells were investigated for delivering functional anti‐miR‐9 to glioblastoma multiforme cells^81^; communication between MSCs and brain glioblastoma cells can be mediated by exosomes.^82^ Flow cytometry and quantitative PCR (qPCR) indicated that MSC‐derived exosomes, loaded with anti‐mir9, led to the reduction of MDR1 expression in T98G and U87 glioblastoma cells. Delivery of anti‐mir9 increased the sensitivity of these cells towards temozolomide, resulting in increased cancer cell death.^81^

Strategies for loading molecular cargo in exosomes and related efficacies differ based on the chemistry of the loaded molecule. The most common method for loading small‐molecule drugs involves mixing the drug solution with a suspension of exosomes and incubating them at 25–37°C. The drug loading efficiency is typically determined using liquid chromatrography.^83^, ^84^ Loading of DNA onto exosomes using electroporation methods is likely restricted by the size of the exosomes with only large exosomes being capable of carrying large linear/plasmid DNA^85^ The loading efficiencies achieved using electroporation varied between 15 and 30% of those achieved with small molecule drugs and siRNA,^86^, ^87^ Momen‐Heravi et al. were able to achieve up to 55% loading efficiency using electroporation for miRNA molecules.^88^ Smyth et al. achieved doxorubicin loading efficiencies of 5% by weight of exosomes using the mixing technique.^89^ Similarly, Sun et al. observed a binding capacity of 2.9 g of curcumin for every gram of exosome using the mixing technique followed by sucrose density gradient centrifugation.^36^ Kim et al. compared different techniques that is, mixing incubation, electroporation, and sonication for loading the drug paclitaxel into exosomes and observed 1.5, 5, and 29% loading efficiencies, respectively.^90^ In comparison, Yang et al. were able achieve 5% loading efficiency of siRNA in cationic lipopolymers with an encapsulation efficiency of 70%.^91^ and Cao et al. obtained 33% loading efficiency in calcium phosphate nanoparticles.^92^

Taken together, these studies indicate the potential utility of exosomes in drug and nucleic acid delivery. The choice of cells from which exosomes are isolated, yield of exosome vesicles, cargo loading procedures, selection of targeting biomolecules (e.g., peptides) on the surface, biodistribution, and immune response are key factors for consideration for exosome‐mediated delivery in future translational applications. Loading of small‐molecule drugs can be efficient, although there is room for improvement in case of loading DNA/siRNA.

## 5 Biomarkers

Exosomes play a vital role in cell‐cell communication by directly engaging with surface ligands and/or by transferring their contents between cells.^93^, ^94^, ^95^, ^96^ Presence of exosomal RNA was implicated as evidence for horizontal transfer of genetic information between various cell types.^97^, ^98^, ^99^ Exosomes are thought to also transfer cellular mRNA as well as microRNA which indicates that tumor exosomes are functional and could suppress mRNA that codes for signal transduction components within T‐cells.^100^, ^101^ The RNA population in tumor‐secreted exosomes includes microRNA and it is possible that this exosomal microRNA reflects the parental tumor signature. As a result, microRNA expression profiling can be useful as a diagnostic tool in diseases, including some cancers which lack definitive molecular biomarkers.

**Figure 5:**
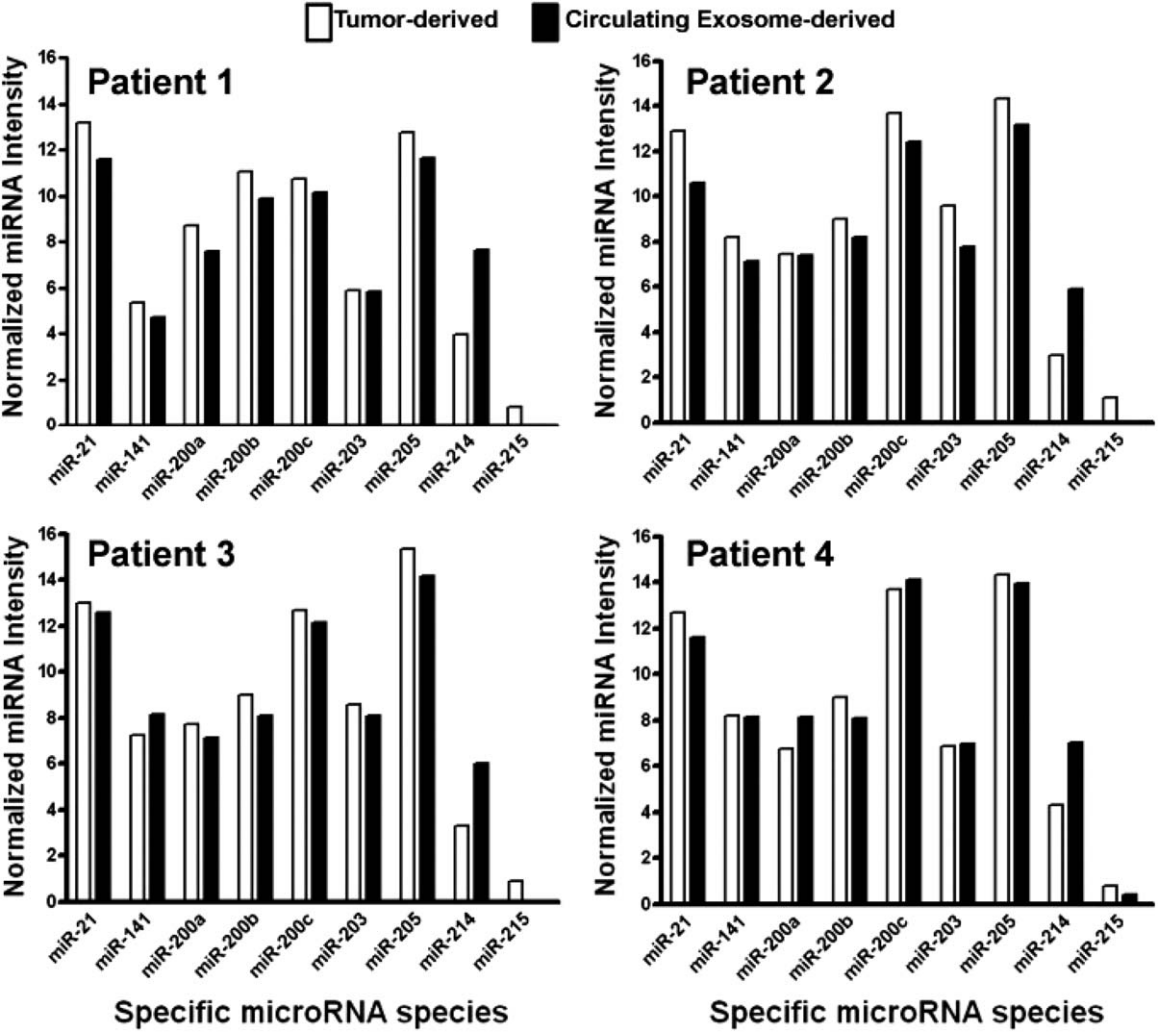
miRNA biomarker expression levels in tumor and exosome‐derived samples showing comparable levels of miRNA between the two sample types. Adapted from Ref. 104

Exosome levels can be slightly elevated in benign tumors and highly elevated in cancerous patients as compared to normal controls; exosomes were isolated from the sera of patients/control subjects using magnetic activated cell sorting. Approximately, 175 different miRNA were found to be similar between tumor cells and exosomes. The up‐regulated miRNA profile from exosomes also matched up‐regulated miRNA profiles in ovarian cancer patients at different stages of the disease. However, this approach was not able to distinguish between different stages of cancer.^102^, ^103^, ^104^, ^105^ Expression of miRNA in circulating tumor‐derived exosomes derived from lung adenocarcinoma patients were similar to that seen in primary tumors, indicating the potential use of these tumor‐derived exosomes as biomarkers. In the future, it may be possible to analyze miRNA from circulating exosomes obviating the need for, or at least complementing, tumor biopsy samples^10^ in applications related to detecting disease, monitoring response to therapy, and investigating cancer recurrence.

The mRNA expression of two distinct biomarkers, PCA‐3 and TMPRSS2, is found in exosomes and can be used to provide a direct link to the incidence of prostate cancer.^106^ Proteins extracted from urinary exosomes can be of potential use in diagnostics of urinary tract diseases,^45^ and prostate and bladder cancers. Eight urinary exosomal proteins were identified as biomarkers when patients with prostate, bladder cancer cells, and healthy cells were compared.^107^ It has been reported in many cases that exosomal miRNA are prospective biomarkers for renal fibrosis and cardiovascular diseases.^108^, ^109^, ^110^

Potential advantages of using exosomes as biomarkers includes the ability to reduce the use of invasive surgery for monitoring disease. Exosomes can be isolated from serum samples and have been successfully used for biomarker detection in ovarian, lung and pancreatic cancers.^111^, ^112^, ^113^ Exosomal miRNA has also been found to be useful in distinguishing between pancreatic carcinomas from benign pancreatic tumors and chronic pancreatitis^113^ and in detection of miRNA (miR‐34A) responsible for conferring drug resistance to prostate cancer.^41^ Exosomal miRNA appear to be stable and can undergo multiple freeze‐thaw cycles, variations in pH and heating without undergoing degradation or loss of expression levels; the miRNA profile is also highly specific to cancer/tumor tissue.^113^ However, a key drawback of using exosomes as biomarkers markers is that they cannot determine the severity of the disease because the miRNA levels can be identical during different stages of the disease as shown by Taylor et al. for ovarian cancer and Rabinowitz et al. in the case of lung cancer.^104^, ^112^ The potential of using exosomal markers for clinical diagnostics needs to be further investigated in depth because various exosomal components including lipids, proteins and miRNAs can be promising in disease diagnostics.

**Figure 6:**
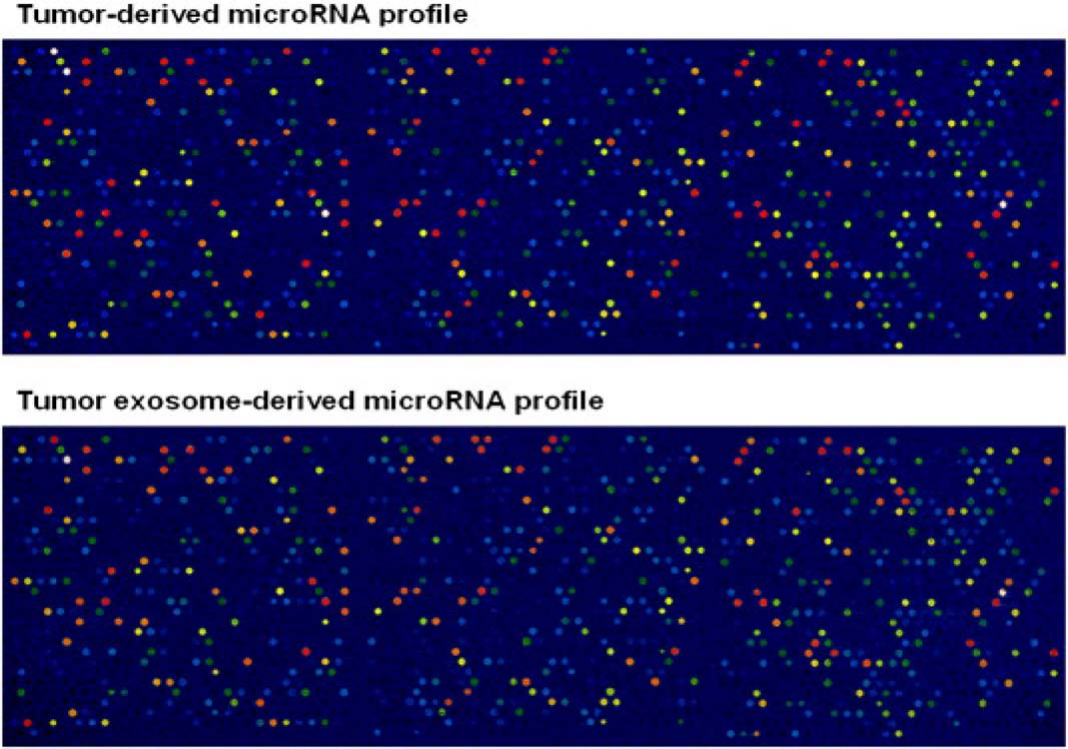
miRNA profile comparison between tumor and circulating exosomes. Adapted from Ref. 104

## 6 Anticancer vaccines

Exosomes derived from specific sites in the body can be promising candidates for anti‐cancer vaccines since they can present antigens against that specific type of cancer. Exosomes can be isolated from three sites of origin ascites (ascite‐derived exosomes or AEX), dendritic cells (dendritic cell‐derived exosomes or DEX), and tumors (tumor‐derived exosomes or TEX).^114^, ^115^, ^116^, ^117^ The antigens in these exosomes can elicit an immune response via MHC class I or class II molecules on CD8^+^ or CD4^+^ T cells. These exosomal MHC class I/II molecules on exosomes are likely used for communication with specific cell types.^118^, ^119^ Melanoma antigens in DEXs were used to prime cytotoxic T lymphocytes in order to elicit an immune response through the Mart1 specific pathway in patients with high‐grade melanoma.^21^, ^120^ In a different study, exosomes were isolated from four different pancreatic cancer cell lines, SOJ‐6, BxPC‐3, MiaPaCa‐2, and Panc‐1. Exosomes derived from SOJ‐6 and BxPC‐3 cells were more effective at reducing growth human pancreatic adenocarcinoma cells compared to those derived from MiaPaCa‐2 and Panc‐1 cells; regulation of Hes‐1 protein via downregulation of the Notch‐1 signaling pathway and activation of mitochondria‐dependent apoptosis pathway played a key role in the cell ablation efficacy.^121^

Dendritic cell‐derived exosomes were shown to elicit Natural Killer (NK) cell responses following intradermal injection in C57BL/6 mice. Exosomes isolated from normal volunteers were primed with melanoma associated antigens MAGE3.A1 and MAGE3.DP04, and were used as vaccines in stage IIIb and stage IV melanoma patients. The treatment resulted in a regression of the cancer; while no changes in cytotoxic T‐lymphocyte levels were observed, the level of NK cell increased with treatment. However, the NK cells obtained from patients that responded to the DEX treatment were more effective at killing K562 cells in culture compared to those obtained from nonresponders.^115^, ^122^

These studies indicate potential applications of exosomes in cancer immunotherapy. However, exosomes generally need mature DCs to elicit a T‐cell response, since antigens present on exosomes need to be taken up by the dendritic cells before they can activate T‐lymphocytes. This can be avoided by the use of adjuvants in some cases, which can allow exosomes to directly prime T‐lymphocytes. In addition, the reliance on an intact immune system can restrict their use in immunocompromised or immunosuppressed patients. However, this approach can elicit an immune response in already developed cancer which can induce regression of the tumor.^122^ This approach may also find less resistance from the suppressive nature of the tumor microenvironment,^116^, ^117^, ^122^, ^123^, ^124^, ^125^, ^126^ which can be a significant advantage.

**Figure 7:**
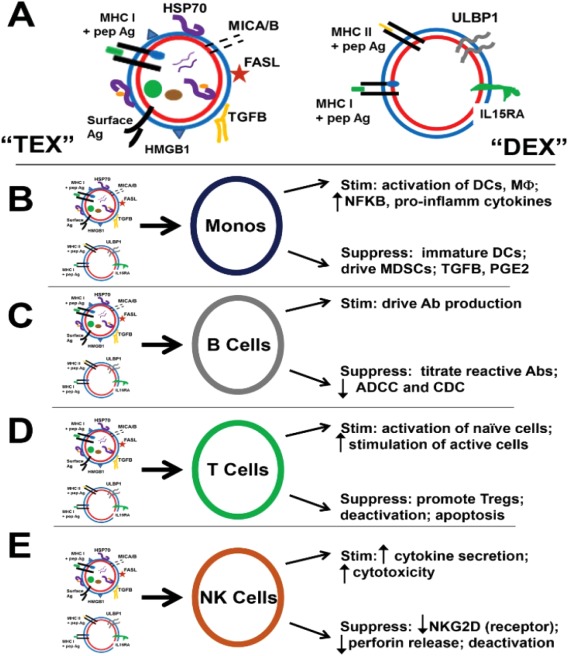
Interactions of dendritic cell‐derived exosomes and tumor‐derived exosomes with inflammatory cells. Reproduced with permission from Creative Commons. Adapted from Ref. 117

## 7 Future directions

The potential for exosomes in the field of drug delivery is significant owing to their ability to selectively express proteins like tetraspanins which may allow for cell targeting. It will be necessary to obtain highly pure formulations of exosomes with low amounts of protein aggregates and other microvascular particles. Optimization of ultracentrifugation coupled with density gradient centrifugation may offer one route toward obtaining enriched populations of exosomes. Further advancements that enhance the loading of drug/nucleic acid are necessary for effective delivery of these therapeutic molecules. Improvements in analytical methods and advances in biomarker discovery can facilitate the use of exosomes in disease detection.

## 8 Conclusions

Exosomes are starting to gather attention in cancer therapeutics and diagnostics, with several applications in drug delivery, tumor immunotherapy, and diagnostic biomarkers. Their unique strengths include enhanced passive targeting due to small size, indigenous nature, and the ability to cross biological barriers. However, the cumbersome nature of the methods required for isolation/purification, inability to distinguish between different cancer stages, and incomplete understanding of their impact on the immune system are some of the current limitations with this technology. It is anticipated that sophisticated engineering and detailed clinical studies that address these limitations will lead to the translation of exosome‐based technologies in the future.

## References

[1] Walther W , Stein U. Viral vectors for gene transfer. Drugs. 2000;60 (2):249–271. 10983732 10.2165/00003495-200060020-00002

[2] Ochman H , Lawrence JG , Groisman EA. Lateral gene transfer and the nature of bacterial innovation. Nature. 2000;405(6784):299–304. 10830951 10.1038/35012500

[3] El Andaloussi S , Mager I , Breakefield XO , Wood MJA. Extracellular vesicles: biology and emerging therapeutic opportunities. Nat Rev Drug Discov. 2013;12(5):347–357. 23584393 10.1038/nrd3978

[4] Kooijmans SAA , Vader P , van Dommelen SM , van Solinge WW , Schiffelers RM. Exosome mimetics: a novel class of drug delivery systems. Int J Nanomedicine. 2012;7:1525–1541. 22619510 10.2147/IJN.S29661 PMC3356169

[5] Lee Y , EL Andaloussi S , Wood MJA. Exosomes and microvesicles: extracellular vesicles for genetic information transfer and gene therapy. Hum Mol Genet. 2012;21(R1):R125–R134. 22872698 10.1093/hmg/dds317

[6] Schiller M , Bekeredjian‐Ding I , Heyder P , Blank N , Ho AD , Lorenz HM. Autoantigens are translocated into small apoptotic bodies during early stages of apoptosis. Cell Death Differ. 2008;15(1):183–191. 17932498 10.1038/sj.cdd.4402239

[7] Simpson RJ , Jensen SS , Lim JWE. Proteomic profiling of exosomes: current perspectives. Proteomics. 2008;8(19):4083–4099. 18780348 10.1002/pmic.200800109

[8] Lin J , Li J , Huang B , et al. Exosomes: novel biomarkers for clinical diagnosis. Sci World J. 2015;2015:8. 10.1155/2015/657086 PMC4322857 25695100

[9] Sokolova V , Ludwig A‐K , Hornung S , et al. Characterisation of exosomes derived from human cells by nanoparticle tracking analysis and scanning electron microscopy. Colloids Surf B: Biointerfaces. 2011;87(1):146–150. 21640565 10.1016/j.colsurfb.2011.05.013

[10] van den Boorn JG , Daßler J , Coch C , Schlee M , Hartmann G. Exosomes as nucleic acid nanocarriers. Adv Drug Deliv Rev. 2013;65(3):331–335. 22750807 10.1016/j.addr.2012.06.011

[11] Simons M , Raposo G. Exosomes – vesicular carriers for intercellular communication. Curr Opin Cell Biol. 2009;21(4):575–581. 19442504 10.1016/j.ceb.2009.03.007

[12] Batista BS , Eng WS , Pilobello KT , Hendricks‐Muñoz KD , Mahal LK. Identification of a conserved glycan signature for microvesicles. J Proteome Res. 2011;10(10):4624–4633. 21859146 10.1021/pr200434y PMC3443565

[13] Kalani A , Tyagi A , Tyagi N. Exosomes: mediators of neurodegeneration, neuroprotection and therapeutics. Mol Neurobiol. 2014;49(1):590–600. 23999871 10.1007/s12035-013-8544-1 PMC3951279

[14] Pant S , Hilton H , Burczynski ME. The multifaceted exosome: biogenesis, role in normal and aberrant cellular function, and frontiers for pharmacological and biomarker opportunities. Biochem Pharmacol. 2012;83(11):1484–1494. 22230477 10.1016/j.bcp.2011.12.037 PMC7110994

[15] Lai RC , Yeo RWY , Tan KH , Lim SK. Exosomes for drug delivery—a novel application for the mesenchymal stem cell. Biotechnol Adv. 2013;31(5):543–551. 22959595 10.1016/j.biotechadv.2012.08.008

[16] van Niel G , Porto‐Carreiro I , Simoes S , Raposo G. Exosomes: a common pathway for a specialized function. J Biochem. 2006;140(1):13–21. 16877764 10.1093/jb/mvj128

[17] Schorey JS , Bhatnagar S. Exosome function: from tumor immunology to pathogen biology. Traffic. 2008;9(6):871–881. 18331451 10.1111/j.1600-0854.2008.00734.x PMC3636814

[18] Raposo G , Nijman HW , Stoorvogel W , et al. B lymphocytes secrete antigen‐presenting vesicles. J Exp Med. 1996;183(3):1161–1172. 8642258 10.1084/jem.183.3.1161 PMC2192324

[19] Raposo G , Tenza D , Mecheri S , Peronet R , Bonnerot C , Desaymard C. Accumulation of major histocompatibility complex class II molecules in mast cell secretory granules and their release upon degranulation. Mol Biol Cell. 1997;8(12):2631–2645. 9398681 10.1091/mbc.8.12.2631 PMC25733

[20] Zitvogel L , Regnault A , Lozier A , et al. Eradication of established murine tumors using a novel cell‐free vaccine: dendritic cell derived exosomes. Nat Med. 1998;4(5):594–600. 9585234 10.1038/nm0598-594

[21] Wolfers J , Lozier A , Raposo G , et al. Nat Med. 2001;7(3):297–303. 11231627 10.1038/85438

[22] Blanchard N , Lankar D , Faure F , et al. TCR activation of human T cells induces the production of exosomes bearing the TCR/CD3/ζ complex. J Immunol. 2002;168(7):3235–3241. 11907077 10.4049/jimmunol.168.7.3235

[23] Escola J‐M , Kleijmeer MJ , Stoorvogel W , Griffith JM , Yoshie O , Geuze HJ. Selective enrichment of tetraspan proteins on the internal vesicles of multivesicular endosomes and on exosomes secreted by human B‐lymphocytes. J Biol Chem. 1998;273(32):20121–20127. 9685355 10.1074/jbc.273.32.20121

[24] Lobb RJ , Becker M , Wen Wen S , Wong CSF , Wiegmans AP , Leimgruber A , Möller A. Optimized exosome isolation protocol for cell culture supernatant and human plasma. J Extracell Vesicles. 2015;4:27031. 10.3402/jev.v4.27031 PMC4507751 26194179

[25] Rabesandratana H , Toutant J‐P , Reggio H , Vidal M. Decay‐accelerating factor (CD55) and membrane inhibitor of reactive lysis (CD59) are released within exosomes during in vitro maturation of reticulocytes. Blood. 1998;91(7):2573–2580. 9516159

[26] Clayton A , Court J , Navabi H , et al. Analysis of antigen presenting cell derived exosomes, based on immuno‐magnetic isolation and flow cytometry. J Immunol Methods. 2001;247(1‐2):163–174. 11150547 10.1016/s0022-1759(00)00321-5

[27] Théry C , Boussac M , Véron P , Ricciardi‐Castagnoli P , Raposo G , Garin J , Amigorena S. Proteomic analysis of dendritic cell‐derived exosomes: a secreted subcellular compartment distinct from apoptotic vesicles. J Immunol. 2001;166(12):7309–7318. 11390481 10.4049/jimmunol.166.12.7309

[28] Skokos D , Le Panse S , Villa I , et al. Mast cell‐dependent B and T lymphocyte activation is mediated by the secretion of immunologically active exosomes. J Immunol. 2001;166(2):868–876. 11145662 10.4049/jimmunol.166.2.868

[29] Van Niel G , Raposo G , Candalh C , et al. Intestinal epithelial cells secrete exosome‐like vesicles. Gastroenterology. 2001;121(2):337–349. 11487543 10.1053/gast.2001.26263

[30] Théry C , Regnault A , Garin J , et al. Molecular characterization of dendritic cell‐derived exosomes. J Cell Biol. 1999;147(3):599–610. 10545503 10.1083/jcb.147.3.599 PMC2151184

[31] Azmi AS , Bao B , Sarkar FH. Exosomes in cancer development, metastasis and drug resistance: a comprehensive review. Cancer Metastasis Rev. 2013;32:0–10. 10.1007/s10555-013-9441-9 PMC3843988 23709120

[32] Braicu C , Tomuleasa C , Monroig P , Cucuianu A , Berindan‐Neagoe I , Calin GA. Exosomes as divine messengers: are they the Hermes of modern molecular oncology[quest]. Cell Death Differ. 2015;22(1):34–45. 25236394 10.1038/cdd.2014.130 PMC4262777

[33] Vlassov AV , Magdaleno S , Setterquist R , Conrad R. Exosomes: current knowledge of their composition, biological functions, and diagnostic and therapeutic potentials. Biochim Biophys Acta. 2012;1820(7):940–948. 22503788 10.1016/j.bbagen.2012.03.017

[34] Wu Y. Melanoma exosomes deliver a complex biological payload that upregulates PTPN11 to suppress T lymphocyte function. Pigment Cell Melanoma Res. 2017;30:203–218. 27930879 10.1111/pcmr.12564 PMC5360477

[35] Yang T , Martin P , Fogarty B , et al. Exosome delivered anticancer drugs across the blood‐brain barrier for brain cancer therapy in Danio Rerio. Pharm Res. 2015;32(6):2003–2014. 25609010 10.1007/s11095-014-1593-y PMC4520542

[36] Sun D , Zhuang X , Xiang X , et al. A novel nanoparticle drug delivery system: the anti‐inflammatory activity of curcumin is enhanced when encapsulated in exosomes. Mol Ther. 2010;18(9):1606–1614. 20571541 10.1038/mt.2010.105 PMC2956928

[37] Chen TS , Arslan F , Yin Y , et al. Enabling a robust scalable manufacturing process for therapeutic exosomes through oncogenic immortalization of human ESC‐derived MSCs. J Transl Med. 2011;9:47‐47. 21513579 10.1186/1479-5876-9-47 PMC3100248

[38] Timmers L , Lim SK , Hoefer IE , et al. Human mesenchymal stem cell‐conditioned medium improves cardiac function following myocardial infarction. Stem Cell Res. 2011;6(3):206–214. 21419744 10.1016/j.scr.2011.01.001

[39] Arslan F , Lai RC , Smeets MB , et al. Mesenchymal stem cell‐derived exosomes increase ATP levels, decrease oxidative stress and activate PI3K/Akt pathway to enhance myocardial viability and prevent adverse remodeling after myocardial ischemia/reperfusion injury. Stem Cell Res. 2013;10(3):301–312. 23399448 10.1016/j.scr.2013.01.002

[40] André F , Chaput N , Schartz NEC , et al. Exosomes as potent cell‐free peptide‐based vaccine. I. Dendritic cell‐derived exosomes transfer functional MHC class I/peptide complexes to dendritic cells. J Immunol. 2004;172(4):2126–2136. 14764678 10.4049/jimmunol.172.4.2126

[41] Corcoran C , Rani S , O'Driscoll L. miR‐34a is an intracellular and exosomal predictive biomarker for response to docetaxel with clinical relevance to prostate cancer progression. Prostate. 2014;74(13):1320–1334. 25053345 10.1002/pros.22848

[42] Chen X , Ba Y , Ma L , et al. Characterization of microRNAs in serum: a novel class of biomarkers for diagnosis of cancer and other diseases. Cell Res. 2008;18:997–1006. 18766170 10.1038/cr.2008.282

[43] Kosaka N , Iguchi H , Ochiya T. Circulating microRNA in body fluid: a new potential biomarker for cancer diagnosis and prognosis. Cancer Sci. 2010;101:2087–2092. 20624164 10.1111/j.1349-7006.2010.01650.x PMC11159200

[44] Brase JC , Wuttig D , Kuner R , Sültmann H. Serum microRNAs as non‐invasive biomarkers for cancer. Mol Cancer. 2010;9:1–9. 21110877 10.1186/1476-4598-9-306 PMC3002336

[45] Zhou H , Cheruvanky A , Hu X , et al. Urinary exosomal transcription factors, a new class of biomarkers for renal disease. Kidney Int. 2008;74(5):613–621. 18509321 10.1038/ki.2008.206 PMC2562924

[46] Van Deun J , Mestdagh P , Sormunen R , et al. The impact of disparate isolation methods for extracellular vesicles on downstream RNA profiling. J Extracellular Vesicles. 2014;3:24858. 10.3402/jev.v3.24858 PMC4169610 25317274

[47] Momen‐Heravi F , Balaj L , Alian S , et al. Current methods for the isolation of extracellular vesicles. Biol Chem. 2013;394:1253. 23770532 10.1515/hsz-2013-0141 PMC7075462

[48] Chen C , Skog J , Hsu C‐H , et al. Microfluidic isolation and transcriptome analysis of serum microvesicles. Lab Chip. 2010;10(4):505–511. 20126692 10.1039/b916199f PMC3136803

[49] Escrevente C , Keller S , Altevogt P , Costa J. Interaction and uptake of exosomes by ovarian cancer cells. BMC Cancer. 2011;11:108‐108. 21439085 10.1186/1471-2407-11-108 PMC3072949

[50] Jeppesen DK , Nawrocki A , Jensen SG , et al. Quantitative proteomics of fractionated membrane and lumen exosome proteins from isogenic metastatic and nonmetastatic bladder cancer cells reveal differential expression of EMT factors. Proteomics. 2014;14(6):699–712. 24376083 10.1002/pmic.201300452

[51] Østergaard O , Nielsen CT , Iversen LV , Jacobsen S , Tanassi JT , Heegaard NHH. Quantitative proteome profiling of normal human circulating microparticles. J Proteome Res. 2012;11(4):2154–2163. 22329422 10.1021/pr200901p

[52] Greening DW , Xu R , Ji H , Tauro BJ , Simpson RJ. A protocol for exosome isolation and characterization: evaluation of ultracentrifugation, density‐gradient separation, and immunoaffinity capture methods In: Posch A, ed. Proteomic Profiling: Methods and Protocols. New York, NY: Springer; 2015:179–209. 10.1007/978-1-4939-2550-6_15 25820723

[53] Théry C , Amigorena S , Raposo G , Clayton A. Isolation and characterization of exosomes from cell culture supernatants and biological fluids In: Bonifacino JS, Harford JB, Lippincott‐Schwartz J, Yamada KM, eds. Current Protocols in Cell Biology. Hoboken, NJ: John Wiley & Sons, Inc; 2001. 10.1002/0471143030.cb0322s30 18228490

[54] Purushothaman A , Bandari SK , Liu J , Mobley JA , Brown EE , Sanderson RD. Fibronectin on the surface of myeloma cell‐derived exosomes mediates exosome‐cell interactions. J Biol Chem. 2016;291(4):1652–1663. 26601950 10.1074/jbc.M115.686295 PMC4722448

[55] Estelles A , Sperinde J , Roulon T , et al. Exosome nanovesicles displaying G protein‐coupled receptors for drug discovery. Int J Nanomed. 2007;2(4):751–760. PMC2676799 18203441

[56] van Balkom BWM , Eisele AS , Pegtel DM , Bervoets S , Verhaar MC. Quantitative and qualitative analysis of small RNAs in human endothelial cells and exosomes provides insights into localized RNA processing, degradation and sorting. J Extracell Vesicles. 2015;4:26760 26027894 10.3402/jev.v4.26760 PMC4450249

[57] Li M , Zeringer E , Barta T , Schageman J , Cheng A , Vlassov AV. Analysis of the RNA content of the exosomes derived from blood serum and urine and its potential as biomarkers. Philos Trans R Soc B Biol Sci. 2014;369(1652):20130502. 10.1098/rstb.2013.0502 PMC4142023 25135963

[58] Guescini M , Genedani S , Stocchi V , Agnati LF. Astrocytes and glioblastoma cells release exosomes carrying mtDNA. J Neural Transm (Vienna). 2009;117(1):1–4. 19680595 10.1007/s00702-009-0288-8

[59] Balaj L , Lessard R , Dai L , et al. Tumour microvesicles contain retrotransposon elements and amplified oncogene sequences. Nat Comms. 2011;2:180. 10.1038/ncomms1180 PMC3040683 21285958

[60] Thakur BK , Zhang H , Becker A , et al. Double‐stranded DNA in exosomes: a novel biomarker in cancer detection. Cell Res. 2014;24(6):766–769. 24710597 10.1038/cr.2014.44 PMC4042169

[61] Pugholm LH , Revenfeld ALS , Søndergaard EKL , Jørgensen MM. Antibody‐based assays for phenotyping of extracellular vesicles. BioMed Res Int. 2015;2015:524817. 26770974 10.1155/2015/524817 PMC4681819

[62] Zarovni N , Corrado A , Guazzi P , et al. Integrated isolation and quantitative analysis of exosome shuttled proteins and nucleic acids using immunocapture approaches. Methods. 2015;87:46–58. 26044649 10.1016/j.ymeth.2015.05.028

[63] Mincheva‐Nilsson L , Baranov V , Nagaeva O , Dehlin E. Isolation and characterization of exosomes from cultures of tissue explants and cell lines In: Colgan JE, Bierer BE, Margulies DH, Shevach EM, Strober W, eds. Current Protocols in Immunology. Hoboken, NJ: John Wiley & Sons, Inc; 2001. 10.1002/cpim.17 27801511

[64] Nordin JZ , Lee Y , Vader P , et al. Ultrafiltration with size‐exclusion liquid chromatography for high yield isolation of extracellular vesicles preserving intact biophysical and functional properties. Nanomedicine. 2015;11(4):879–883. 25659648 10.1016/j.nano.2015.01.003

[65] Gercel‐Taylor C , Atay S , Tullis RH , Kesimer M , Taylor DD. Nanoparticle analysis of circulating cell‐derived vesicles in ovarian cancer patients. Anal Biochem. 2012;428(1):44–53. 22691960 10.1016/j.ab.2012.06.004

[66] Rider MA , Hurwitz SN , Meckes DG. ExtraPEG: a polyethylene glycol‐based method for enrichment of extracellular vesicles. Sci Rep. 2016;6:23978. 27068479 10.1038/srep23978 PMC4828635

[67] Davies RT , Kim J , Jang SC , Choi E‐J , Gho YS , Park J. Microfluidic filtration system to isolate extracellular vesicles from blood. Lab Chip. 2012;12(24):5202–5210. 23111789 10.1039/c2lc41006k

[68] Taylor DD , Zacharias W , Gercel‐Taylor C. Exosome isolation for proteomic analyses and RNA profiling In Simpson JR, Greening WD, eds. Serum/Plasma Proteomics: Methods and Protocols. Totowa, NJ: Humana Press; 2011:235‐246. 10.1007/978-1-61779-068-3_15 21468952

[69] Taylor DD , Shah S. Methods of isolating extracellular vesicles impact down‐stream analyses of their cargoes. Methods. 2015;87:3–10. 25766927 10.1016/j.ymeth.2015.02.019

[70] Hong P , Koza S , Bouvier ESP. Size‐exclusion chromatography for the analysis of protein biotherapeutics and their aggregates. J Liquid Chromatogr Relat Technol. 2012;35(20):2923–2950. 10.1080/10826076.2012.743724 PMC3556795 23378719

[71] Wang Z , Wu H‐j , Fine D , et al. Ciliated micropillars for the microfluidic‐based isolation of nanoscale lipid vesicles. Lab Chip. 2013;13(15):2879–2882. 23743667 10.1039/c3lc41343h PMC3740541

[72] Baranyai T , Herczeg K , Onódi Z , et al. Isolation of exosomes from blood plasma: qualitative and quantitative comparison of ultracentrifugation and size exclusion chromatography methods. PloS One. 2015;10(12):e0145686. 26690353 10.1371/journal.pone.0145686 PMC4686892

[73] Witwer KW , Buzás EI , Bemis LT , et al., Standardization of sample collection, isolation and analysis methods in extracellular vesicle research. *J Extracell Vesicles*. 2013;2:20360. doi:10.3402/jev.v2i0.20360 10.3402/jev.v2i0.20360 PMC3760646 24009894

[74] Wu Y , Deng W , Klinke DJ. Exosomes: improved methods to characterize their morphology, RNA content, and surface protein biomarkers. Analyst. 2015;140(19):6631–6642. 26332016 10.1039/c5an00688k PMC4986832

[75] Zhuang X , Xiang X , Grizzle W , et al. Treatment of brain inflammatory diseases by delivering exosome encapsulated anti‐inflammatory drugs from the nasal region to the brain. Mol Ther. 2011;19(10):1769–1779. 21915101 10.1038/mt.2011.164 PMC3188748

[76] Candolfi M , Curtin JF , Stephen Nichols W , et al. Intracranial glioblastoma models in preclinical neuro‐oncology: neuropathological characterization and tumor progression. J Neurooncol. 2007;85(2):133–148. 17874037 10.1007/s11060-007-9400-9 PMC2384236

[77] Haney MJ , Klyachko NL , Zhao Y , et al. Exosomes as drug delivery vehicles for Parkinson's disease therapy. J Control Release. 2015;207:18–30. 25836593 10.1016/j.jconrel.2015.03.033 PMC4430381

[78] Haney MJ , Klyachko NL , Zhao Y , et al. Exosomes as drug delivery vehicles for Parkinson's disease therapy. J Controlled Release. 2015;207:18–30. 10.1016/j.jconrel.2015.03.033 PMC4430381 25836593

[79] Munagala R , Aqil F , Jeyabalan J , Gupta RC. Bovine milk‐derived exosomes for drug delivery. Cancer Lett. 2016;371(1):48–61. 26604130 10.1016/j.canlet.2015.10.020 PMC4706492

[80] Alvarez‐Erviti L , Seow Y , Yin H , Betts C , Lakhal S , Wood MJA. Delivery of siRNA to the mouse brain by systemic injection of targeted exosomes. Nat Biotechnol. 2011;29(4):341–345. 21423189 10.1038/nbt.1807

[81] Munoz JL , Bliss SA , Greco SJ , Ramkissoon SH , Ligon KL , Rameshwar P. Delivery of functional anti‐miR‐9 by mesenchymal stem cell‐derived exosomes to glioblastoma multiforme cells conferred chemosensitivity. Mol Ther Nucleic Acids. 2013;2:e126. 24084846 10.1038/mtna.2013.60 PMC4027430

[82] Biancone L , Bruno S , Deregibus MC , Tetta C , Camussi G. Therapeutic potential of mesenchymal stem cell‐derived microvesicles. Nephrol Dial Transplant. 2012;27(8):3037–3042. 22851627 10.1093/ndt/gfs168

[83] Zhuang X , Xiang X , Grizzle W , et al. Treatment of brain inflammatory diseases by delivering exosome encapsulated anti‐inflammatory drugs from the nasal region to the brain. Mol Ther. 2011;19(10):1769–1779. 21915101 10.1038/mt.2011.164 PMC3188748

[84] Jang SC , Kim OY , Yoon CM , et al. Bioinspired exosome‐mimetic nanovesicles for targeted delivery of chemotherapeutics to malignant tumors. ACS Nano. 2013;7(9):7698–7710. 24004438 10.1021/nn402232g

[85] Lamichhane TN , Raiker RS , Jay SM. Exogenous DNA loading into extracellular vesicles via electroporation is size‐dependent and enables limited gene delivery. Mol Pharmaceutics. 2015;12(10):3650–3657. 10.1021/acs.molpharmaceut.5b00364 PMC4826735 26376343

[86] Banizs AB , Huang T , Dryden K , Berr SS , Stone JR , Nakamoto RK , Shi W , He J. In vitro evaluation of endothelial exosomes as carriers for small interfering ribonucleic acid delivery. Int J Nanomed. 2014;9:4223–4230. 10.2147/IJN.S64267 PMC4159392 25214786

[87] Tian Y , Li S , Song J , et al. A doxorubicin delivery platform using engineered natural membrane vesicle exosomes for targeted tumor therapy. Biomaterials. 2014;35(7):2383–2390. 24345736 10.1016/j.biomaterials.2013.11.083

[88] Momen‐Heravi F , Bala S , Bukong T , Szabo G. Exosome‐mediated delivery of functionally active miRNA‐155 inhibitor to macrophages. Nanomedicine. 2014;10(7):1517–1527. 24685946 10.1016/j.nano.2014.03.014 PMC4180003

[89] Smyth T , Kullberg M , Malik N , Smith‐Jones P , Graner MW , Anchordoquy TJ. Biodistribution and delivery efficiency of unmodified tumor‐derived exosomes. J Controlled Release. 2015;199:145–155. 10.1016/j.jconrel.2014.12.013 PMC4441346 25523519

[90] Kim MS , Haney MJ , Zhao Y , et al. Development of exosome‐encapsulated paclitaxel to overcome MDR in cancer cells. Nanomedicine. 2016;12(3):655–664. 26586551 10.1016/j.nano.2015.10.012 PMC4809755

[91] Yang X‐Z , Dou S , Sun T‐M , Mao C‐Q , Wang H‐X , Wang J. Systemic delivery of siRNA with cationic lipid assisted PEG‐PLA nanoparticles for cancer therapy. J Control Release. 2011;156(2):203–211. 21839126 10.1016/j.jconrel.2011.07.035

[92] Cao X , Deng W , Wei Y , et al. Encapsulation of plasmid DNA in calcium phosphate nanoparticles: stem cell uptake and gene transfer efficiency. Int J Nanomed. 2011;6:3335–3349. 10.2147/IJN.S27370 PMC3252680 22229000

[93] Polgar J , Matuskova J , Wagner DD. The P‐selectin, tissue factor, coagulation triad. J Thromb Haemost. 2005;3(8):1590–1596. 16102023 10.1111/j.1538-7836.2005.01373.x

[94] Barry OP , Praticò D , Savani RC , FitzGerald GA. Modulation of monocyte‐endothelial cell interactions by platelet microparticles. J Clin Invest. 1998;102(1):136–144. 9649567 10.1172/JCI2592 PMC509075

[95] Rozmyslowicz T , Majka M , Kijowski J , et al. Platelet‐and megakaryocyte‐derived microparticles transfer CXCR4 receptor to CXCR4‐null cells and make them susceptible to infection by X4‐HIV. Aids. 2003;17(1):33–42. 12478067 10.1097/00002030-200301030-00006

[96] Camussi G , Deregibus MC , Bruno S , Cantaluppi V , Biancone L. Exosomes/microvesicles as a mechanism of cell‐to‐cell communication. Kidney Int. 2010;78(9):838–848. 20703216 10.1038/ki.2010.278

[97] Baj‐Krzyworzeka M , Szatanek R , Węglarczyk K , et al. Tumour‐derived microvesicles carry several surface determinants and mRNA of tumour cells and transfer some of these determinants to monocytes. Cancer Immunol Immunother. 2006;55(7):808–818. 16283305 10.1007/s00262-005-0075-9 PMC11030663

[98] Deregibus MC , Cantaluppi V , Calogero R , et al. Endothelial progenitor cell–derived microvesicles activate an angiogenic program in endothelial cells by a horizontal transfer of mRNA. Blood. 2007;110(7):2440–2448. 17536014 10.1182/blood-2007-03-078709

[99] Yuan A , Farber EL , Rapoport AL , et al. Transfer of microRNAs by embryonic stem cell microvesicles. PloS One. 2009;4(3):e4722. 19266099 10.1371/journal.pone.0004722 PMC2648987

[100] Valenti R , Huber V , Filipazzi P , et al. Human tumor‐released microvesicles promote the differentiation of myeloid cells with transforming growth factor‐β–mediated suppressive activity on T lymphocytes. Cancer Res. 2006;66(18):9290–9298. 16982774 10.1158/0008-5472.CAN-06-1819

[101] Whiteside TL. Immune modulation of T‐cell and NK (natural killer) cell activities by TEXs (tumour‐derived exosomes). Biochm Soc Trans. 2013;41(1):245–251. 10.1042/BST20120265 PMC3721347 23356291

[102] Henderson MC , Azorsa DO. The genomic and proteomic content of cancer cell‐derived exosomes. Front Oncol. 2012;2:38. 22649786 10.3389/fonc.2012.00038 PMC3355967

[103] Melo SA , Sugimoto H , O'Connell Joyce T , et al. Cancer exosomes perform cell‐independent MicroRNA biogenesis and promote tumorigenesis. Cancer Cell. 26(5):707–721. 25446899 10.1016/j.ccell.2014.09.005 PMC4254633

[104] Taylor DD , Gercel‐Taylor C. MicroRNA signatures of tumor‐derived exosomes as diagnostic biomarkers of ovarian cancer. Gynecol Oncol. 2008;110(1):13–21. 18589210 10.1016/j.ygyno.2008.04.033

[105] Rosell R , Wei J , Taron M. Circulating MicroRNA signatures of tumor‐derived exosomes for early diagnosis of non–small‐cell lung cancer. Clin Lung Cancer. 2009;10(1):8–9. 19289365 10.3816/CLC.2009.n.001

[106] Mincheva‐Nilsson L , Baranov V. Cancer exosomes and NKG2D receptor–ligand interactions: impairing NKG2D‐mediated cytotoxicity and anti‐tumour immune surveillance. Semin Cancer Biol. 2014;28:24–30. 24602822 10.1016/j.semcancer.2014.02.010

[107] Smalley DM , Sheman NE , Nelson K , Theodorescu D. Isolation and identification of potential urinary microparticle biomarkers of bladder cancer. J Proteome Res. 2008;7(5):2088–2096. 18373357 10.1021/pr700775x

[108] Kuwabara Y , Ono K , Horie T , et al. Increased MicroRNA‐1 and MicroRNA‐133a levels in serum of patients with cardiovascular disease indicate myocardial damage. Circulation. 2011;4(4):446–454. 21642241 10.1161/CIRCGENETICS.110.958975

[109] Lv L‐L , Cao Y‐H , Pan M‐M , et al. CD2AP mRNA in urinary exosome as biomarker of kidney disease. Clin Chim Acta. 2014;428:26–31. 24144866 10.1016/j.cca.2013.10.003

[110] Chen J‐F , Mandel EM , Thomson JM , et al. The role of microRNA‐1 and microRNA‐133 in skeletal muscle proliferation and differentiation. Nat Genet. 2006;38(2):228–233. 16380711 10.1038/ng1725 PMC2538576

[111] Resnick KE , Alder H , Hagan JP , Richardson DL , Croce CM , Cohn DE. The detection of differentially expressed microRNAs from the serum of ovarian cancer patients using a novel real‐time PCR platform. Gynecol Oncol. 2009;112:55–59. 18954897 10.1016/j.ygyno.2008.08.036

[112] Rabinowits G , Gerçel‐Taylor C , Day JM , Taylor DD , Kloecker GH. Exosomal MicroRNA: a diagnostic marker for lung cancer. Clin Lung Cancer. 2009;10(1):42–46. 19289371 10.3816/CLC.2009.n.006

[113] Que R , Ding G , Chen J , Cao L. Analysis of serum exosomal microRNAs and clinicopathologic features of patients with pancreatic adenocarcinoma. World J Surg Onc. 2013;11(1):219. 10.1186/1477-7819-11-219 PMC3766671 24007214

[114] Whiteside TL. Tumor‐derived exosomes and their role in tumor‐induced immune suppression. Vaccines. 2016;4(4):35. 10.3390/vaccines4040035 PMC5192355 27775593

[115] Viaud S , Terme M , Flament C , et al. Dendritic cell‐derived exosomes promote natural killer cell activation and proliferation: a role for NKG2D ligands and IL‐15Rα. PLoS One. 2009;4(3):e4942. 19319200 10.1371/journal.pone.0004942 PMC2657211

[116] Runz S , Keller S , Rupp C , et al. Malignant ascites‐derived exosomes of ovarian carcinoma patients contain CD24 and EpCAM. Gynecol Oncol. 2007;107(3):563–571. 17900673 10.1016/j.ygyno.2007.08.064

[117] Kunigelis KE , Graner MW. The dichotomy of tumor exosomes (TEX) in cancer immunity: is it all in the ConTEXt? Vaccines (Basel). 2015;3(4):1019–1051. 26694473 10.3390/vaccines3041019 PMC4693230

[118] Lynch S , Santos SG , Campbell EC , et al. Novel MHC class I structures on exosomes. J Immunol. 2009;183(3):1884–1891. 19596992 10.4049/jimmunol.0900798

[119] LeBrasseur N. MHC and antigen in exosomes. J Cell Biol. 2007;179(1):4–4.

[120] Escudier B , Dorval T , Chaput N , et al. Vaccination of metastatic melanoma patients with autologous dendritic cell (DC) derived‐exosomes: results of thefirst phase I clinical trial. J Transl Med. 2005;3(1):1. 15740633 10.1186/1479-5876-3-10 PMC554765

[121] Ristorcelli E , Beraud E , Mathieu S , Lombardo D , Verine A. Essential role of Notch signaling in apoptosis of human pancreatic tumoral cells mediated by exosomal nanoparticles. Int J Cancer. 2009;125(5):1016–1026. 19405120 10.1002/ijc.24375

[122] Tan A , De La Peña H , Seifalian AM. The application of exosomes as a nanoscale cancer vaccine. Int J Nanomedicine. 2010;5:889–900. 21116329 10.2147/IJN.S13402 PMC2990382

[123] Simona F , Laura S , Simona T , Riccardo A. Contribution of proteomics to understanding the role of tumor‐derived exosomes in cancer progression: state of the art and new perspectives. Proteomics. 2013;13(10‐11):1581–1594. 23401131 10.1002/pmic.201200398

[124] Keller S , König A‐K , Marmé F , et al. Systemic presence and tumor‐growth promoting effect of ovarian carcinoma released exosomes. Cancer Lett. 2009;278(1):73–81. 19188015 10.1016/j.canlet.2008.12.028

[125] André F , Schartz NEC , Chaput N , et al. Tumor‐derived exosomes: a new source of tumor rejection antigens. Vaccine. 2002;20:A28–A31., *Supplement 4*, 12477425 10.1016/s0264-410x(02)00384-5

[126] Kharaziha P , Ceder S , Li Q , Panaretakis T. Tumor cell‐derived exosomes: a message in a bottle. Biochim Biophys Acta. 2012;1826(1):103–111. 22503823 10.1016/j.bbcan.2012.03.006

